# Celastrol attenuates streptozotocin-induced diabetic cardiomyopathy in mice by inhibiting the ACE / Ang II / AGTR1 signaling pathway

**DOI:** 10.1186/s13098-023-01159-x

**Published:** 2023-09-12

**Authors:** Xuyong Zhao, Bingwu Huang, Jianhua Zhang, Wenjun Xiang, Ning Zhu

**Affiliations:** 1 Department of Cardiology, The Wenzhou Third Clinical Institute, The Third Affiliated Hospital of Shanghai University, Wenzhou Medical University, Wenzhou People’s Hospital, No. 299 Guan Road, Wenzhou, Zhejiang Province People’s Republic of China; 2 Department of Pathology, The Wenzhou Third Clinical Institute, The Third Affiliated Hospital of Shanghai University, Wenzhou Medical University, Wenzhou People’s Hospital, Wenzhou, China; 3 Department of Anesthesiology and Perioperative Medicine, The Second Affiliated Hospital, Yuying Children’s Hospital of Wenzhou Medical University, Wenzhou, China

**Keywords:** ACE signaling pathway, Apoptosis, Celastrol, Diabetic cardiomyopathy, Oxidative stress

## Abstract

**Background:**

Heart failure is closely correlated with diabetic cardiomyopathy (DCM) and can lead to mortality. Celastrol has long been utilized for the treatment of immune and inflammatory disorders. However, whether celastrol would exert protective effects on DCM has not been determined. This work aimed to explore the protective actions of celastrol on DCM and unravel the underlying mechanisms involved.

**Methods:**

A DCM model was constructed in mice by intraperitoneal administration of streptozotocin. ELISA and echocardiography were performed to examine myocardial injury markers and cardiac function, respectively. Morphological changes and fibrosis were assessed using H&E staining and Masson’s staining. Inflammatory cytokines and fibrotic markers were detected by ELISA and RT-PCR. Endothelial nitric oxide synthase, apoptosis, and reactive oxygen species were detected by microscopic staining. Network pharmacology approaches, molecular docking analysis, ELISA, and Western blot were used for mechanism studies.

**Results:**

Celastrol alleviated diabetes-induced cardiac injury and remodeling. Celastrol also suppressed diabetes-induced production of inflammatory cytokines and reactive oxygen species, as well as cardiomyocyte apoptosis. The cardioprotective effects of celastrol were associated with its inhibition on the angiotensin-converting enzyme / angiotensin II / angiotensin II receptor type 1 signaling pathway.

**Conclusion:**

Celastrol exhibits significant potential as an effective cardioprotective drug for DCM treatment. The underlying mechanisms can be attributed to the blockage of celastrol on the angiotensin-converting enzyme signaling pathway.

**Supplementary Information:**

The online version contains supplementary material available at 10.1186/s13098-023-01159-x.

## Introduction

Diabetic cardiomyopathy (DCM) refers to cardiomyopathy induced by diabetes but unaccompanied by valvular disease, coronary artery disease, and hypertension. Heart failure (HF) is a commonly observed complication of diabetes that contributes to unfavorable outcomes in patients diagnosed with DCM [1]. The conventional approaches to intensive blood glucose control have demonstrated limited effectiveness in reducing the risk of HF and improving prognosis [2]. The traditional treatment for HF remains consistent for patients with DCM and those without. In addition, there is currently no targeted therapy available for HF associated with DCM. Sodium-glucose cotransporter 2 inhibitors have emerged as a promising strategy for treating DCM patients with HF [3, 4]. Their beneficial effects, however, are independent of glucose-lowering properties. Hence, it is imperative to discover and develop novel drugs that can improve cardiac dysfunction and subsequent HF associated with DCM.

*Tripterygium wilfordii Hook. F.* (TwHF) is a traditional Chinese herb medicine that has gained extensive usage in the treatment of inflammatory and immune disorders (i.e., rheumatoid arthritis). Celastrol, the major bioactive ingredient of TwHF’s root [5], is well known for its ability to reduce inflammation [6]. It has also demonstrated therapeutic potential in the management of neurodegenerative diseases and cancers [7]. Celastrol has also exhibited therapeutic effects on a number of metabolic diseases, such as diabetes and obesity, in both cell and animal models [8, 9]. The mechanisms underlying the beneficial effects of celastrol are mainly associated with the TLR3 / NLRP3 inflammasome, the NF-κB and JAK2 / STAT3 pathways [10–12]. It is well known that TLR3 / NLRP3 inflammasome, the NF-κB and JAK2 / STAT3 pathways mediated inflammatory diseases [13–15]. However, whether celastrol would exert protective effects on DCM remains unknown.

**Fig. 1:**
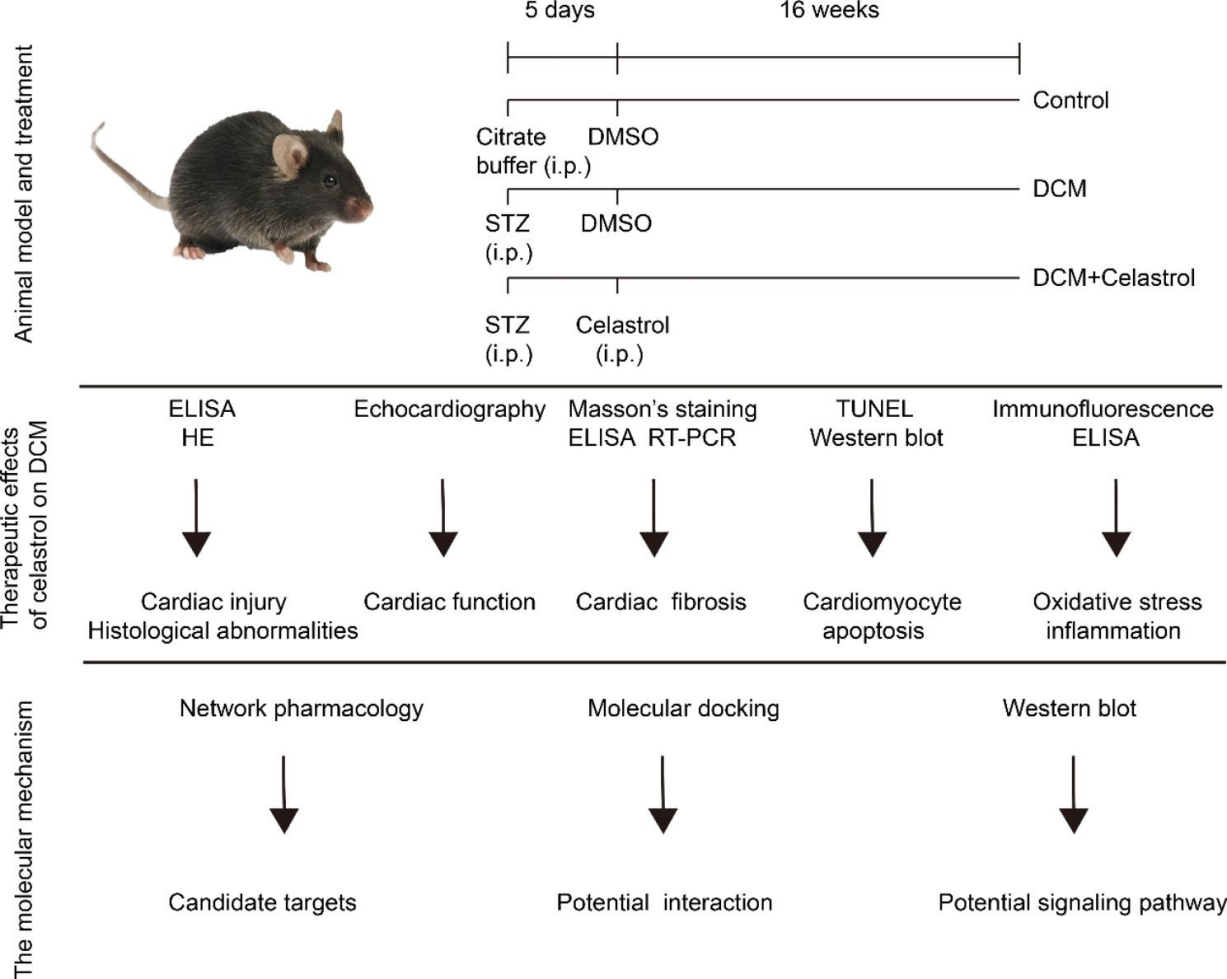
The flowchart for study design

The pharmacological actions of celastrol are considered to arise from its intervention with classic inflammatory signaling pathways. Network pharmacology approaches provide a fundamental strategy for uncovering the mechanisms of action of multi-target drugs [16], during which the data obtained from multiple databases are analyzed and multiple interaction networks are constructed. Computational molecular docking analysis plays an essential role in evaluating the potential of drug binding to its target and deciphering the underlying mechanisms [17]. In this work, we performed animal studies, computational molecular docking analysis, and network pharmacology analysis to examine the impact of celastrol on streptozotocin (STZ)-induced DCM and the molecular mechanisms involved. The flowchart for study design was shown in Fig. 1.

## Methods

### Animal model and treatment

A total of 36 male C57BL/6 mice (weight: 18–22 g, age: 6–7 weeks) were obtained from Slac Animal Co., Ltd. (China). The study procedures were reviewed and approved by the Wenzhou Medical University Animal Policy and Welfare Committee (the approval number: xmsq2022-0704), and the experiments were performed following the National Institutes of Health Guide for the Care and Use of Laboratory Animals. 36 mice were used in the study and 12 mice were in each group Mice were housed under standardized conditions: 12-h light/dark cycle, a humidity level of 50 ± 5%, and a temperature of 20 ± 2 ℃. They were intraperitoneally administered with 50 mg/kg of STZ (S0130, Sigma, USA) or vehicle (citrate buffer) for five consecutive days. 12 mice were served as the control group. Fasting blood glucose levels were recorded seven days after injection, and values higher than 16.7 mmol/L indicated successful establishment of the model. Celastrol (S1290; Selleck Chemicals) was dissolved in DMSO (Sigma). Diabetic mice were randomly assigned into the following groups: DCM and celastrol + DCM. The DCM group were administered with DMSO, while the celastrol + DCM group was treated with 1 mg/kg/2d of celastrol via intraperitoneal injection. Celastrol treatment was maintained for 16 weeks. Blood glucose levels and body weight were recorded every week. Heart tissues were harvested at 16 weeks and either fixed in 4% paraformaldehyde or kept at − 80 °C or for subsequent analysis.

### ELISA

Blood samples from mice were collected via retrobulbar bleeding and left at ambient temperature for half an hour. After centrifugation at ambient temperature for 15 min at 3500 rpm, the serum was obtained and kept at − 80 ℃. The levels of lactate dehydrogenase, angiotensin II (Ang II), and serum creatine kinase MB isoenzyme were determined by ELISA (AssayGenie and Nanjing Jiancheng Bioengineering Research Institute, China). The concentrations of IL-1β, TNF-α, and IL-6, were measured by ELISA (Beyotime and DAKEWE, China).

### Echocardiography

Anesthesia was induced in mice via intraperitoneal administration of pentobarbital at a dosage of 60 mg/kg. Mice were anesthetized by isoflurane inhalation before echocardiography. Noninvasive transthoracic echocardiography was then performed to assess cardiac function. The left ventricular end-systolic diameter (LVESD), ejection fraction (EF), early mitral diastolic wave/late mitral diastolic wave ratio, and left ventricular end-diastolic diameter (LVEDD) were recorded.

### Histopathological analysis

Following sacrifice, heart tissues were harvested from each mouse to assess cardiac remodeling. The tissues were embedded in paraffin and sectioned at a thickness of 4 μm. Cardiac fibrosis and myocardial injury were detected by Masson’s staining and hematoxylin and eosin (H&E) staining, respectively. Cardiac remodeling was assessed by a light microscope (Nikon; 400× magnification).

### RT-PCR

Heart tissues were subjected to total RNA extraction. After reverse transcription, the mRNA levels of type III collagen (COL3) and type I collagen (COL1) were measured by RT-PCR using ABI QuantStudio™ 12 K Flex. The primers are shown in Supplementary Table 1. The expression level of the analyzed transcript was compared to that of GAPDH and normalized to the mean value of the control.

### Reactive oxygen species (ROS) analysis

The 4-µm-thick frozen sections of heart tissues were incubated in ROS solution at 37 °C for half an hour, followed by three rinses with phosphate-buffered saline (PH = 7.4). Cell nuclei were dyed with DAPI for 30 min at ambient temperature, and the fluorescence intensity was examined using fluorescence microscopy (Model: 80i, Nikon, Japan).

### Apoptosis analysis

The terminal deoxyribonucleotide transferase-mediated dUTP nick end-labeling (TUNEL) assay kit (Atagenix, China) was used to detect cardiomyocyte apoptosis. Following dewaxing and rehydrating, 4-µm-thick heart tissue sections were rinsed with phosphate-buffered saline, permeabilized with 0.02 µg/µL of proteinase K, and subjected to TUNEL staining. The percentages of TUNEL-positive cells were calculated using a fluorescence microscope (Olympus, Japan).

### Identification of potential targets associated with the therapeutic effects of celastrol on DCM

The 2D molecular structure and canonical simplified molecular input line entry system of celastrol were retrieved from PubChem (HTTPS://pubchem.ncbi.nlm.nih.gov/). To predict the potential targets of celastrol, the simplified molecular input line entry system was inputted into SwissTargetPrediction (http://www.swisstargetprediction.ch) [18]. The Comparative Toxicogenomics Database [19] and the GeneCards database [20] were used to obtain the candidate targets of DCM. The threshold value was defined as an inference score greater than 30. The duplicate targets of DCM from both databases were removed, while the common targets were retained. Lastly, the Venn diagram was constructed (https://bioinfogp.cnb.csic.es/tools/venny/).

### Protein-protein interaction (PPI) network

The PPI network was built using the STRING database (https://cn.string-db.org/). The relevant data (tsv.) were exported [21] and then imported into Cytoscape (v3.7.1) to develop the network of celastrol against DCM.

### Gene Ontology (GO) and Kyoto Encyclopedia of genes and genomes (KEGG) analysis

WebGestalt (http://www.webgestalt.org) [22] was used for GO and KEGG enrichment analysis that encompasses the biological process, molecular function, and cellular component of the effects of celastrol on DCM. The over-representation analysis method was employed for enrichment analysis. The false discovery rate-adjusted *P*-values were calculated, and a value below 0.05 suggested statistical significance.

### Western blot

RIPA buffer supplemented with phosphatase and protease inhibitors (Beyotime Biotechnology, China) was used for heart tissues homogenization. Total protein extract (30–50 µg) was loaded to 8–12% SDS-PAGE, transferred onto PVDF membranes, blocked by non-fat dry milk (5%), and then stained with designated primary antibodies: angiotensin II receptor type 1 (AGTR1; Proteintech, 1:1000), cleaved-caspase-3 (Cell Signaling Technology, 1:1000), angiotensin-converting enzyme (ACE; Proteintech, 1:1000), Bcl-2 (Proteintech, 1:1000), Bad (Proteintech, 1:1000), and β-actin (Proteintech, 1:2000) at 4 °C for 24 h. Subsequently, the membranes were rinsed with TBST three times and incubated with goat anti-mouse (Proteintech, 1:1000) or goat anti-rabbit IgG (Proteintech, 1:1000) secondary antibody at ambient temperature for 60 min. The fluorescence signal was visualized by the ChemiDoc™ XRS + imaging system (Bio-Rad, USA) and quantified using Image J (v5.0; National Institutes of Health, USA).

### Molecular docking analysis

The 3D chemical structure of celastrol was retrieved from PubChem and subsequently imported into ChemBio3D Ultra (v14) for energy minimization. The crystal structure of ACE, which was acquired from PDB (https://www.rcsb.org), was decorated by removing ligands and water motifs using PyMOL (v2.3). Both the celastrol and ACE structures were imported into AutoDockTools (v1.5.6) for hydrogen addition, charge distribution, and charge calculation. Lastly, docking simulations between celastrol and ACE were performed 10 times using AutoDock Vina (v1.1.2). The search space was size_x:60, size_y: 60 and size_z:60. MM2 was used for energy minimization and binding ability < -5 was considered as the criteria for ideal docking result.

### Statistical analysis

All values are shown as mean ± standard deviation and analyzed by GraphPad Pro5.0 (GraphPad, USA). Comparisons between two groups were analyzed using the *t*-test, while multiple group comparisons were conducted using the one-way ANOVA. A *p*-value below 0.05 indicated statistically significant.

## Results

### Celastrol attenuates cardiac injury in diabetic mice

**Fig. 2:**
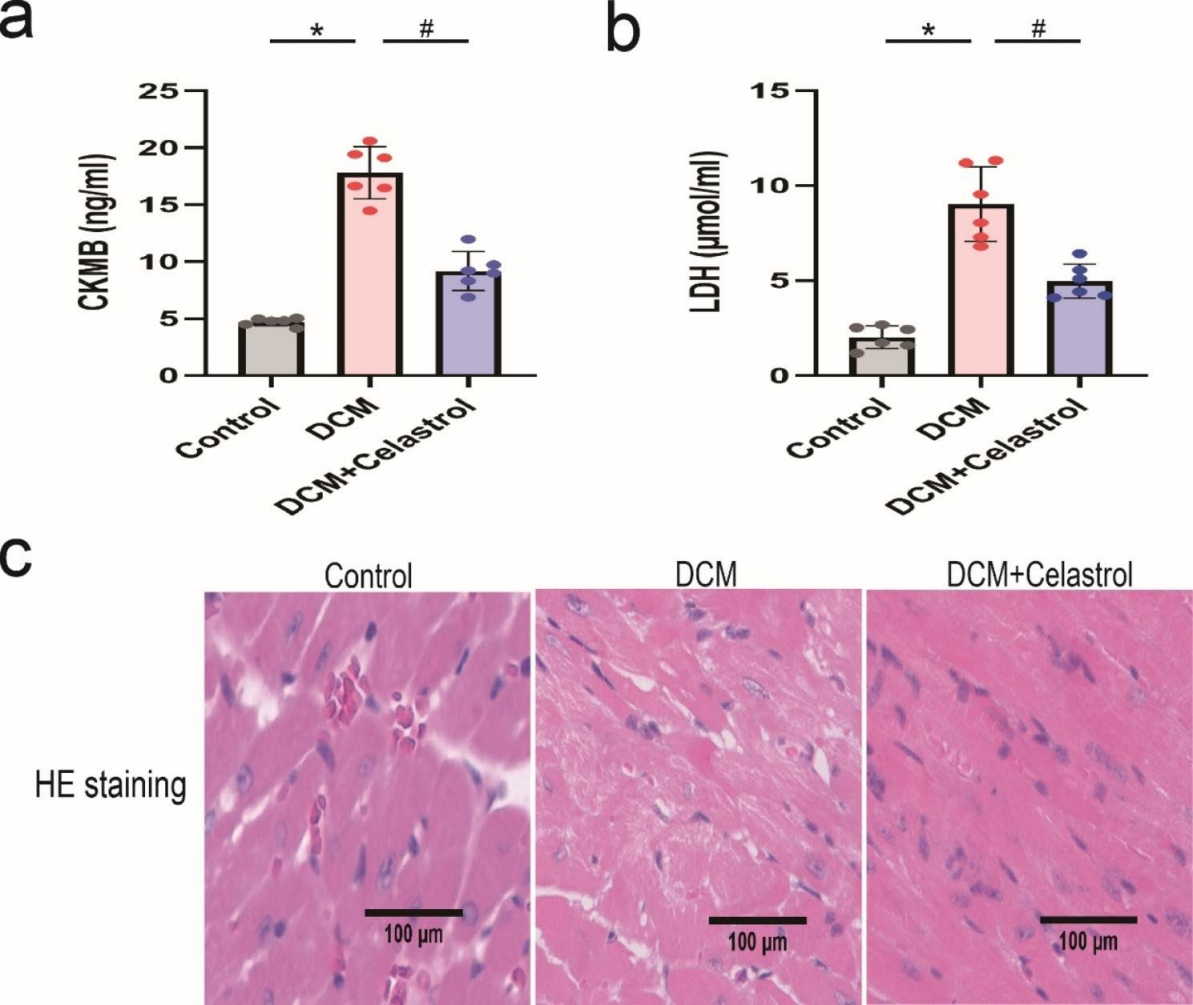
The effects of celastrol on diabetes-induced cardiac injury. **(A, B)** ELISA analysis of heart CK-MB and LDH expression at 16 weeks after STZ injection. **(C)** HE staining of heart tissue at 16 weeks after STZ injection. Magnification, 200×. Data are shown as mean ± SD. n = 6, *P < 0.05 vs. Control; # P < 0.05 vs. DCM + celastrol.

The ELISA results demonstrated that the concentrations of myocardial injury markers, lactate dehydrogenase and serum creatine kinase MB isoenzyme, were increased in the model animals but decreased in diabetic mice treated with celastrol (Fig. 2a, b). H&E staining showed histological abnormalities in the hearts of diabetic mice, while celastrol treatment effectively mitigated these abnormalities (Fig. 2c).

### Celastrol improves cardiac function in diabetic mice

**Fig. 3:**
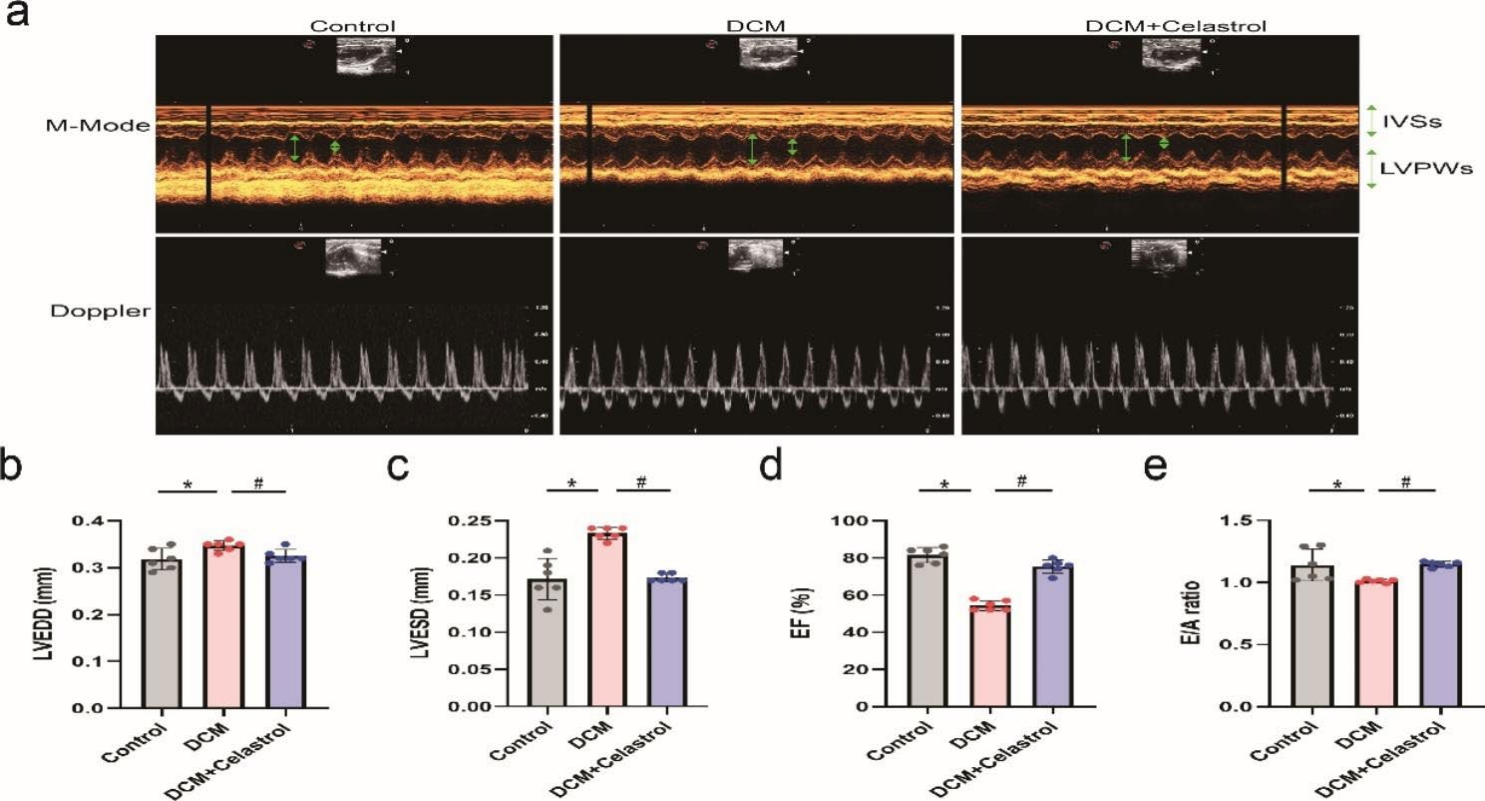
The effects of celastrol on diabetes-induced. **(A)** Representative M-mode and Doppler echocardiography images. **(B-E)** Relative quantification of cardiac function parameters of mice, including left ventricular end-systolic diameter and end-diastolic diameter (LVESD, LVEDD), ejection fraction (EF), and early mitral diastolic wave/late mitral diastolic wave (E/A) ratio. Data are shown as mean ± SD. n = 6, *P < 0.05 vs. Control; # P < 0.05 vs. DCM + celastrol.

To assess cardiac function, we measured LVEDD, LVESD, EF, and FS using echocardiography (Fig. 3a). Mice treated with STZ exhibited notable cardiac diastolic and systolic dysfunction, including elevated LVEDD and LVESD (Fig. 3b, c) and reduced EF and FS (Fig. 3d, e). Celastrol treatment, however, increased EF and FS and reduced LVEDD and LVESD in diabetic mice.

### Celastrol reduces histological abnormalities and cardiac fibrosis in diabetic mice

**Fig. 4:**
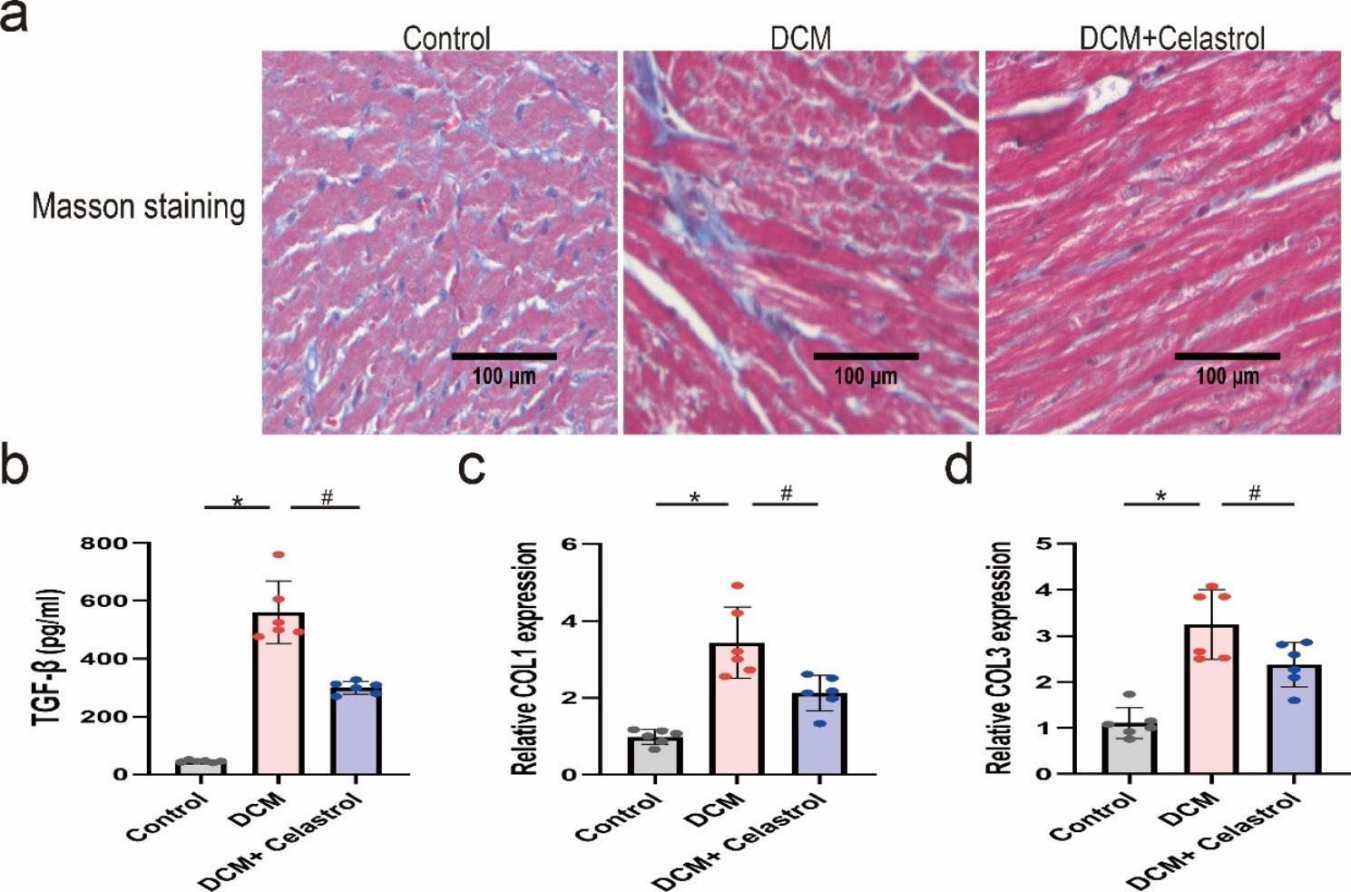
The effects of celastrol on diabetes-induced cardiac fibrosis. **(A)** Representative Masson staining images of the hearts treated by STZ for 16 weeks. Magnification, 200×. **(B)** Quantification of ELISA assay of TGF-β expression in hearts. **(C, D)** Quantification of RT-PCR assay of COL1 and COL3 mRAN level. Data are shown as mean ± SD. n = 6, *P < 0.05 vs. Control; # P < 0.05 vs. DCM + celastrol.

To examine the impact of celastrol on histological alterations and fibrosis in the hearts of diabetic mice, we performed Masson’s staining, ELISA, and RT-PCR analysis to measure fibrotic markers, including TGF-β, COL1, and COL3. Diabetic mice showed serious myocardial fibrosis (Fig. 4a) and upregulated levels of TGF-β, COL1, and COL3 (Fig. 4b–d). However, celastrol treatment ameliorated myocardial fibrosis and downregulated profibrotic markers.

### Celastrol mediates oxidative stress and inflammation induced by diabetes

**Fig. 5:**
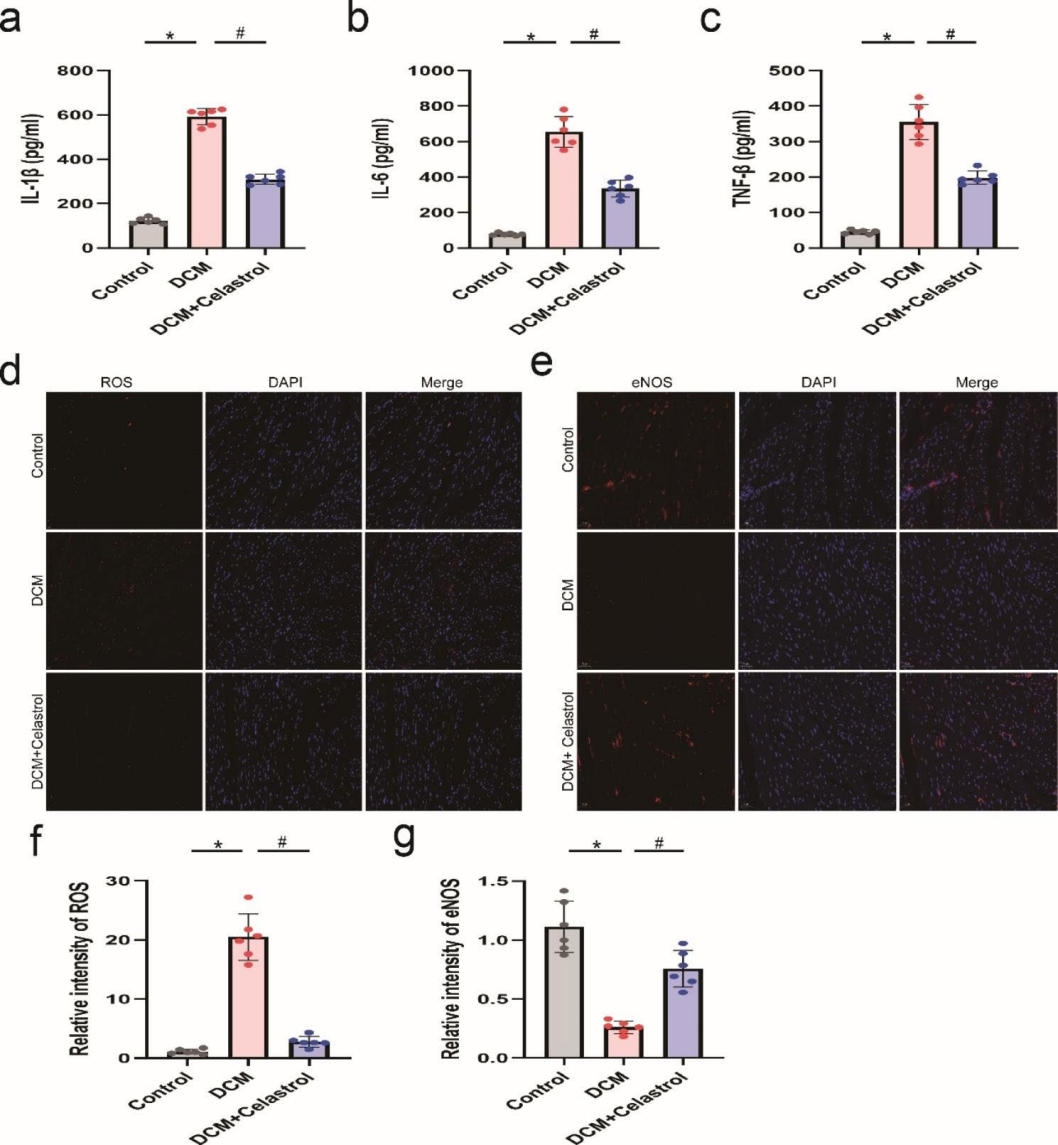
The effects of celastrol on diabetes-induced inflammation and oxidative stress. **(A–C)** Quantification of ELISA assay of IL-1β, IL-6 and TNF-α. **(D, E)** Representative immunofluorescence staining of ROS and eNOS. Scale bar = 20 μm. **(F, G)** Relative quantification of ROS and eNOS. Data are shown as mean ± SD. n = 6, *P < 0.05 vs. Control; # P < 0.05 vs. DCM + celastrol.

In model animals, we observed excessive production of IL-1β, TNF-α, IL-6. However, after celastrol intervention, the levels of these inflammatory cytokines were decreased (Fig. 5a–c). We also explored the impact of celastrol on oxidative stress. Immunofluorescence staining showed increased ROS generation and decreased expression of antioxidant marker endothelial nitric oxide synthase in the hearts of model animals (Fig. 5d-g). Administration with celastrol, however, significantly inhibited the overproduction of ROS and endothelial nitric oxide synthase.

### Celastrol decreases cardiomyocyte apoptosis in diabetic mice

**Fig. 6:**
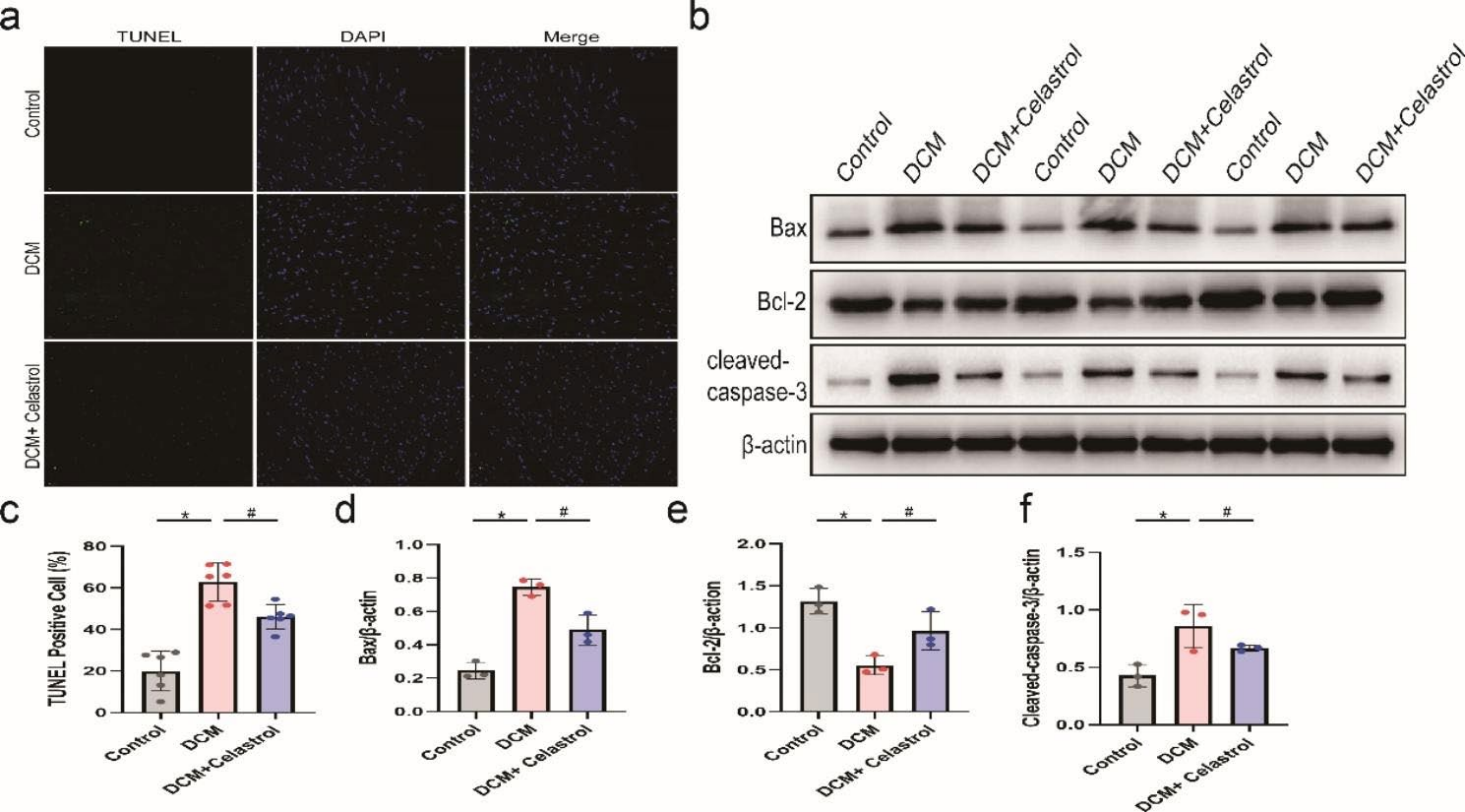
The effects of celastrol on diabetes-induced apoptosis. **(A, C)** Representative TUNEL staining images and relative quantification of TUNEL-positive cell in the hearts treated by STZ for 16 weeks. Scale bar = 20 μm. **(B, D-F)** Representative Western blot images and relative quantification of Bax, Bcl-2 and cleaved-caspase 3 of the hearts treated by STZ for 16 weeks. Data are shown as mean ± SD. n = 6, *P < 0.05 vs. Control; # P < 0.05 vs. DCM + celastrol.

Excessive ROS generation has been considered a key contributor to apoptosis [23]. Additionally, our previous study has also suggested that diabetes may lead to cardiomyocyte apoptosis [24]. Here, TUNEL staining demonstrated a remarkable increase in the quantity of apoptotic cardiomyocytes in mice with diabetes. However, this increase was reversed by celastrol treatment (Fig. 6a, c). Moreover, celastrol reversed the upregulation of cleaved-caspase-3 and Bax/Bal-2 in diabetic mice (Fig. 6b, d-f).

### The core targets of celastrol against DCM and the PPI network

**Fig. 7:**
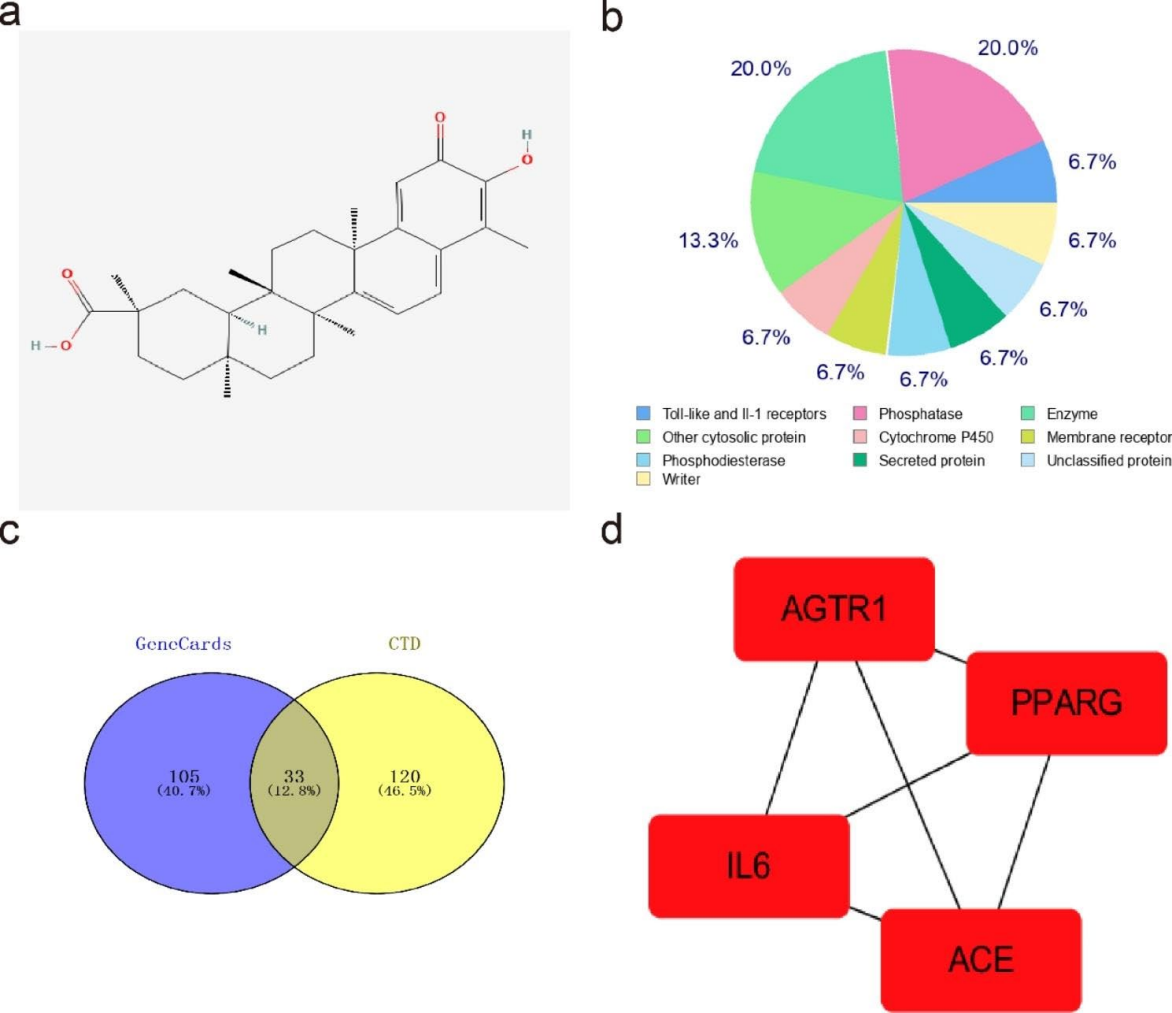
Identifcation of the common potential targets celastrol against DCM. **(A)** The 2D molecular structure of celastrol. **(B)** The distribution map of 100 candidate targets of celastrol collected from the SwissTargetPrediction database. **(C)** Venn diagram of the intersection of DCM from CTD and GeneCards database. **(D)** PPI network of core genes

To further elucidate the mechanisms of action of celastrol in DCM, we performed network pharmacology analysis. From the Comparative Toxicogenomics Database and GeneCards database, a total of 33 common targets related to DCM and 102 targets related to celastrol were identified (Supplementary Table 2) (Fig. 7b, c). Subsequently, four core targets of celastrol against DCM were determined, including ACE, AT1R, peroxisome proliferator-activated receptor gamma, and IL-6. Figure 6d depicts the PPI network of these candidate targets.

#### GO and KEGG analysis

**Fig. 8:**
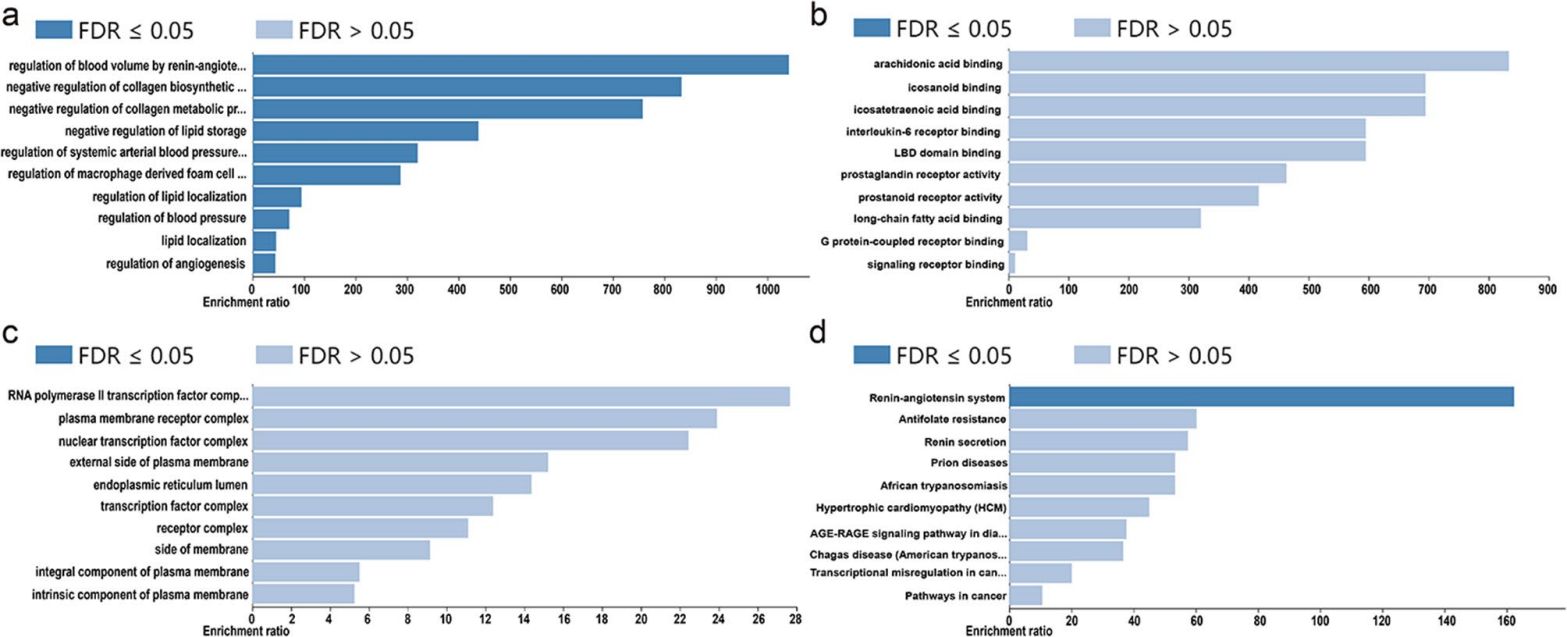
Identifcation of GO and KEGG pathway enrichment. The top 10 signifcantly enriched terms in BP **(A)**, MF **(B)**, and CC **(C)** categories and (B) the top 10 pathway enrichment obtained from WebGestalt website. FDR-adjusted P value < 0.05 indicates statistically significance

To identify the main pathways through which celastrol exerts its effect on DCM, we conducted GO and KEGG enrichment analysis. The data revealed that biological process was primarily related to the regulation of blood volume via the renin-angiotensin pathway. Moreover, the renin-angiotensin-aldosterone system (RAAS) pathway (Fig. 8a) was mainly involved in the protective impact of celastrol on DCM (Fig. 7d).

### Celastrol regulates the ACE / Ang II / AGTR1 pathway in diabetic mice

**Fig. 9:**
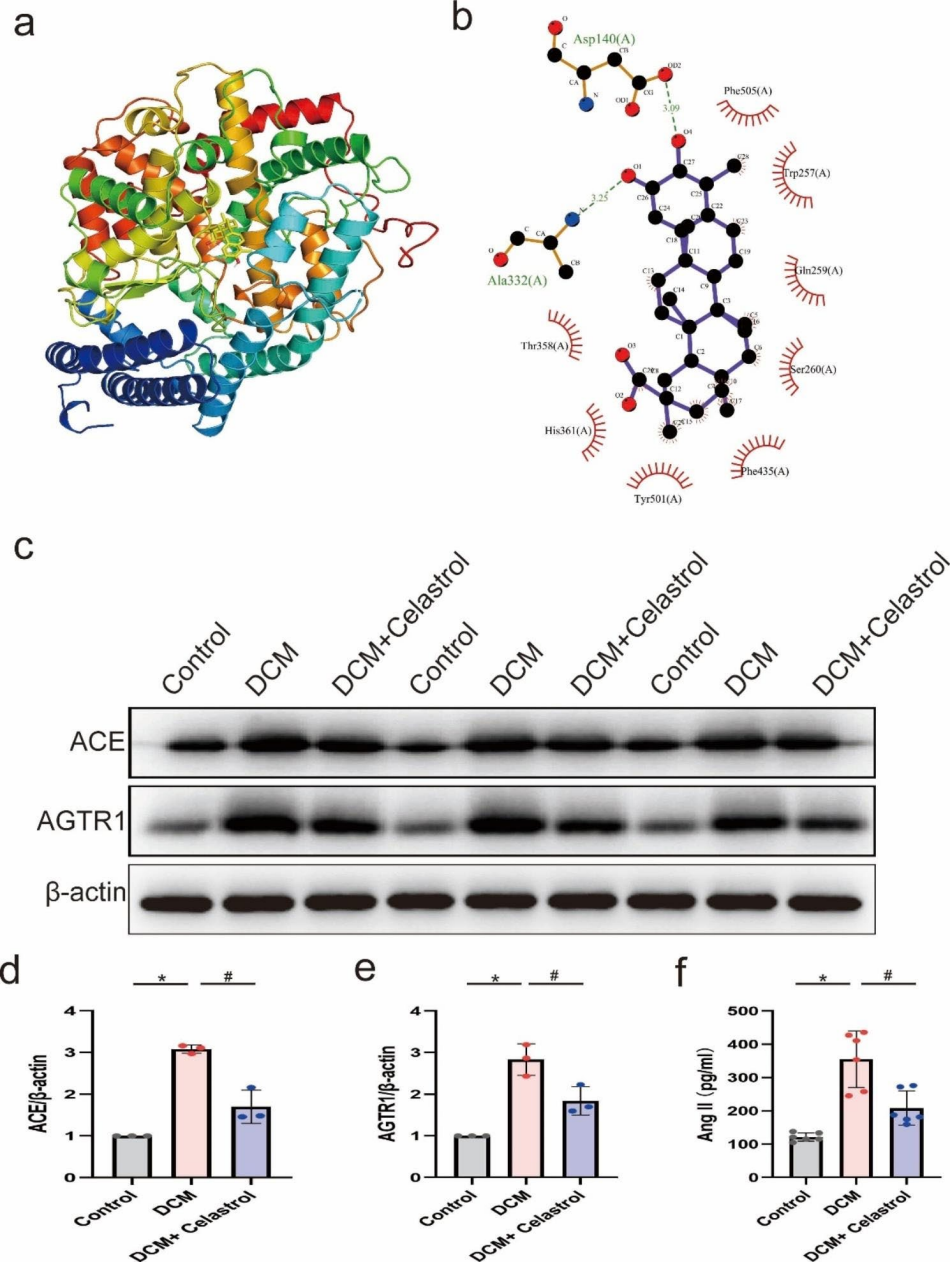
The effects of celastrol on ACE/AngII/AGTR1 signaling pathway in DCM. **(A, B)** The molecular docking simulation of the binding pattern of celastrol with ACE. **(C-E)** Representative Western blot images and relative quantification of ACE and AGTR1, n = 3. **(F)** Quantification of ELISA assay of AngII expression in hearts, n = 6. *P < 0.05 vs. Control; # P < 0.05 vs. DCM + celastrol.

The molecular docking analysis showed that celastrol can bind to the docking pocket of ACE (Fig. 9a, b), with a binding energy of − 10.9 kcal/mol. Celastrol binds to ACE through a binding pocket consisting of Ala332 (3.25Å) and Asp140 (3.09Å) (Fig. 9e). Western blot and ELISA were then conducted to validate the findings of network pharmacology analysis. We found that STZ markedly increased the expression of ACE, Ang II, and AGTR1, while celastrol treatment significantly suppressed the upregulation of these proteins (Fig. 9c-f).

**Fig. 10:**
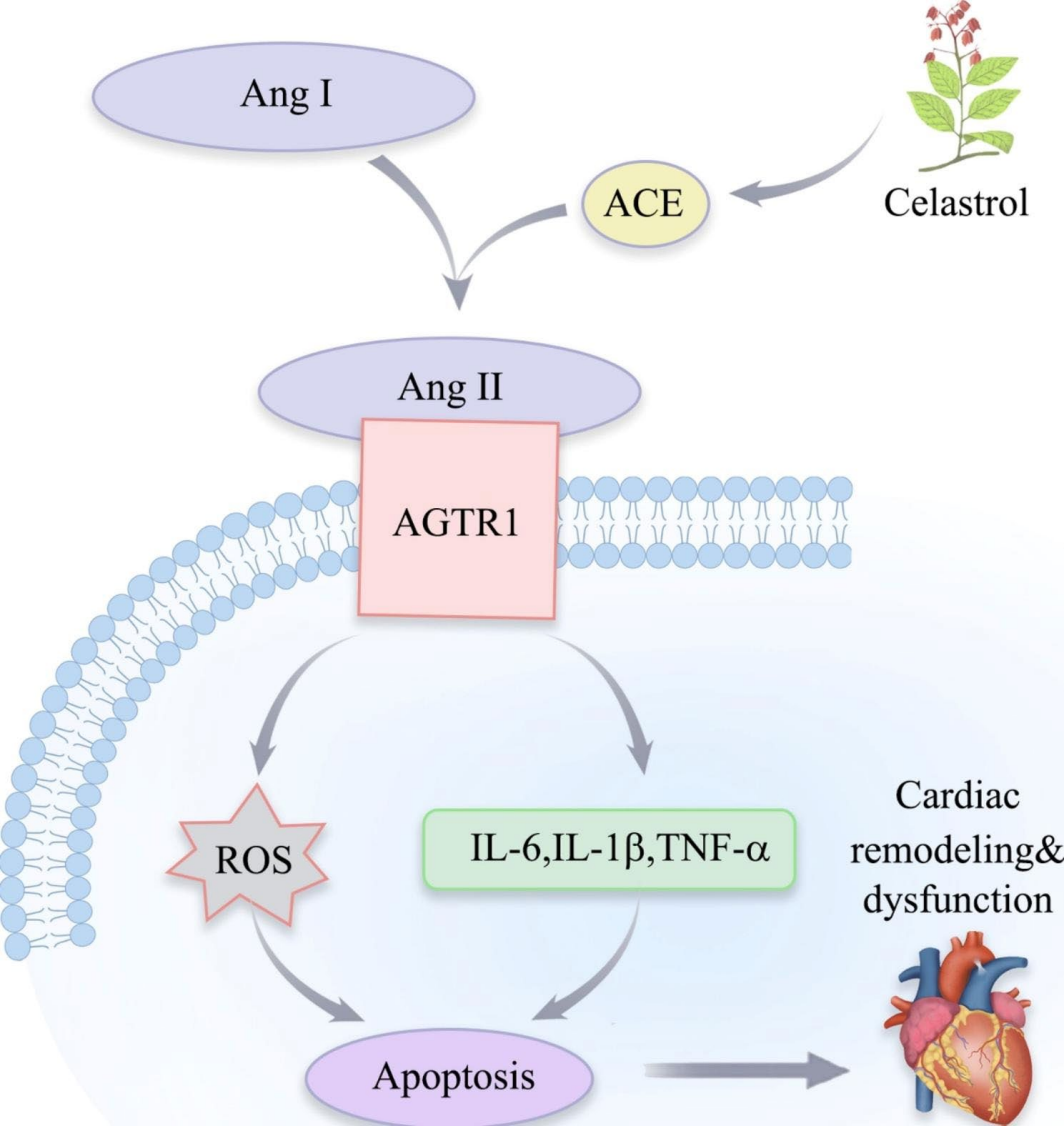
The mechanism of celastrol protecting against DCM.

The mechanisms of the protective effects of celastrol on DCM is shown in Fig. 10.

## Discussion

The goal of the present work was to examine the protective effects of celastrol on DCM and identify the mechanisms involved. To achieve this, an STZ-induced DCM model was established in mice. Cardiac injury, dysfunction, and remodeling were assessed to examine the effects of celastrol on DCM. Additionally, cardiac oxidative stress and apoptosis in mice with diabetes were also examined. Moreover, molecular docking and network pharmacology analyses were performed to elucidate the pharmacological mechanism of celastrol.

The close relationship between HF and diabetes has been widely recognized [25]. Effective therapeutic approaches for DCM or diabetes-related HF are currently limited. The recently developed drug dapagliflozin has demonstrated protective effects that are not necessarily related to the presence of diabetes, suggesting a mechanism of cardioprotective action independent of glucose-lowering properties. Celastrol derived from TwHF has exhibited remarkable therapeutic properties in the treatment of various metabolic disorders [26, 27]. Recent literature has reported that celastrol attenuates cardiac dysfunction and remodeling triggered by transverse aortic constriction myocardial infarction and Ang II [26, 28, 29]. Celastrol has also been considered a therapeutic ingredient for right ventricular failure induced by pulmonary arterial hypertension [30]. In agreement with previous findings, our work demonstrated that celastrol alleviates DCM by improving cardiac injury, remodeling, and dysfunction, suggesting that celastrol may be an effective therapeutic option for DCM.

The complex phenotypes under the pathophysiology of DCM have been well described in previous literature [31]. Aberrant cardiac energy metabolism, oxidative stress, inflammation, cardiomyocyte apoptosis, lipotoxicity, mitochondrial dysfunction pathways, and neurohumoral mechanisms are key factors associated with DCM and HF. Oxidative stress plays an indispensable role in cardiac inflammation and remodeling associated with diabetes, resulting from an imbalance between free radicals and antioxidants [32, 33]. Increasing data have illustrated that excess ROS generation contributes to oxidative stress, subsequently leading to contractile cardiomyocyte apoptosis, cardiac remodeling, and eventually HF [34, 35]. Celastrol was reported to exert cardioprotective effects by modulating inflammation, fibrosis, and apoptosis. In the current study, celastrol suppressed the upregulation of proinflammatory cytokines and induced the production of anti-inflammatory molecules in mice with STZ-induced DCM. In addition, celastrol reduced ROS production and cardiomyocyte apoptosis induced by STZ stimulation. The above findings suggest that celastrol protects against DCM by suppressing inflammation, oxidative stress, and apoptosis.

Network pharmacology analysis offers a systematic approach to evaluate the potential mechanism of multitarget drugs by assessing multiple databases and complex interactions among targets. It has been widely used in investigating key pathways and molecule targets for the treatment of various diseases [36]. In this study, ACE, AGTR1, peroxisome proliferator-activated receptor gamma, and IL-6 were identified as key targets of celastrol for DCM treatment. However, GO and KEGG enrichment analysis implied that the RAAS pathway may play an important role in the effects of celastrol against DCM. Celastrol treatment suppressed the upregulation of ACE, Ang II, and AGTR1 in diabetic mice. Diabetes-induced activation of RAAS in the heart has been observed in both clinical and preclinical settings. Targeting RAAS through pharmacological and gene knockout approaches has proven to be effective in treating DCM in animal models, implying the contribution of the RAAS pathway to DCM [37, 38]. One of the main axes of the RAAS pathway relies on the production of Ang II by ACE, with the AT1 receptor as the effector molecule. Hence, the ACE / Ang II / AGTR1 axis regulates key biological processes in the diabetic heart [39]. The AGTR1 and ACE inhibitors have been frequently used in clinical practice, highlighting the critical role of the ACE / Ang II / AGTR1 signaling pathway. Hyperglycemia activates the RAAS pathway, leading to increased oxidative stress, cardiomyocyte apoptosis, and fibrosis, and ultimately contributing to cardiac dysfunction [40, 41]. In this work, we observed a reduction in oxidative stress, cardiomyocyte apoptosis, inflammation, and fibrosis following the inhibition of the ACE / Ang II / AGTR1 axis. Numerous inflammatory signaling pathways have been implicated as underlying mechanisms in celastrol-mediated effects. As far as we are aware, the targeting of ACE by celastrol has not been previously reported. Here, the result of molecular docking analysis showed celastrol may target ACE. We confirmed that celastrol regulated the ACE / Ang II / AGTR1 signaling pathway by Western blot. Considering that AGTR1 and ACE inhibitors are essential in HF treatment [42, 43], celastrol may be used as a therapeutic agent for HF caused by various etiologies. The novel pharmacological mechanism identified in this study also provides a theoretical basis for the future use of celastrol in the management of other diseases.

### Conclusion and limitation

This study showed that celastrol attenuated cardiac injury, dysfunction, inflammation, fibrosis, apoptosis, and oxidative stress in the DCM model. Notably, the ACE / Ang II / AGTR1 signaling pathway played a significant part in the protective effect of celastrol against DCM. Taken together, our findings provide evidence supporting the therapeutic efficacy of celastrol in the management of DCM and reveal a novel pharmacological mechanism of celastrol. Future research could be conducted to investigate the effects of celastrol on other models of HF and potential side effects. Additional investigations are warranted to explore the inhibitory activity of celastrol on ACE. In addtion, positive control/existing treatment and the concentration gradient were not applied in this study. The targets of celastrol and DCM were obtained from public databases, which may lead to inaccurate results. The protective role of celastrol in the treatment of patients with DCM and HF should evaluated in future study.

### Electronic supplementary material

Below is the link to the electronic supplementary material.

Supplementary Material 1

Supplementary Material 2

Supplementary Material 3

## Data Availability

and statement. The data that support the findings of this study are available from the corresponding author upon reasonable request.

## Abbreviations

DCM: Diabetic cardiomyopathy
HF: Heart failure
TwHF: *Tripterygium wilfordii Hook. F.*
STZ: Streptozotocin
Ang II: Angiotensin II
LVESD: Left ventricular end-systolic diameter
EF: Ejection fraction
LVEDD: Left ventricular end-diastolic diameter
IVSd: intraventricular septum thickness in diastole
LVPWd: LV posterior wall thickness in diastole
H&E: Hematoxylin and eosin
COL3: Type III collagen
COL1: Type I collagen
ROS: Reactive oxygen species
TUNEL: Terminal deoxyribonucleotide transferase-mediated dot nick end-labeling
PPI: Protein-protein interaction
GO: Gene Ontology
KEGG: Kyoto Encyclopedia of Genes and Genomes
AGTR1: Angiotensin II receptor type 1
ACE: Angiotensin-converting enzyme
RAAS: Renin-angiotensin-aldosterone system
