# Supplementary material for: Celastrol attenuates streptozotocin-induced diabetic cardiomyopathy in mice by inhibiting the ACE / Ang II / AGTR1 signaling pathway
Source: Diabetol Metab Syndr. 2023 Sep 12;15:186. doi: 10.1186/s13098-023-01159-x (PMC10496318; doi:10.1186/s13098-023-01159-x)
Supplement: Supplementary file 2 — Supplementary Material 2 [file 13098_2023_1159_MOESM2_ESM.docx]

| **Name** | **Sequence（5’-3’）** | **Size** |
| --- | --- | --- |
| GAPDH | AAGAGGGATGCTGCCCTTAC | 119bp |
|  | ACGGCCAAATCCGTTCACA |  |
| Type I collagen | GGGGCAAGACAGTCATCGAA | 159bp |
|  | GAGGGAACCAGATTGGGGTG |  |
| type III collagen | ACGTAAGCACTGGTGGACAG | 96bp |
|  | GGAGGGCCATAGCTGAACTG |  |
